# Dexamethasone, Cerebrospinal Fluid Matrix Metalloproteinase Concentrations and Clinical Outcomes in Tuberculous Meningitis

**DOI:** 10.1371/journal.pone.0007277

**Published:** 2009-09-30

**Authors:** Justin A. Green, Chau T. H. Tran, Jeremy J. Farrar, Mai T. H. Nguyen, Phu H. Nguyen, Sinh X. Dinh, Nghia D. T. Ho, Chuong V. Ly, Hien T. Tran, Jon S. Friedland, Guy E. Thwaites

**Affiliations:** 1 Department of Infectious Diseases and Immunity, Imperial College London, London, United Kingdom; 2 Oxford University Clinical Research Unit, Hospital for Tropical Diseases, Ho Chi Minh City, Vietnam; 3 Centre for Molecular Microbiology and Infection, Imperial College London, London, United Kingdom; University of Stellenbosch, South Africa

## Abstract

**Background:**

Adjunctive dexamethasone reduces mortality from tuberculous meningitis, but how it produces this effect is not known. Matrix metalloproteinases (MMPs) are important in the immunopathology of many inflammatory CNS diseases thus we hypothesized that that their secretion is important in TBM and might be influenced by dexamethasone.

**Methodology/Principal Findings:**

The kinetics of cerebrospinal fluid (CSF) MMP and tissue inhibitors of MMPs (TIMPs) concentrations were studied in a subset of HIV uninfected adults (n = 37) with TBM recruited to a randomized, placebo-controlled trial of adjuvant dexamethasone. Analysis followed a pre-defined plan. Dexamethasone significantly reduced CSF MMP-9 concentrations in early follow up samples (median 5 days (range 3–8) of treatment), but had no significant influence on other MMPs/TIMPs. Additionally CSF MMP-9 concentration was strongly correlated to concomitant CSF neutrophil count.

**Conclusions/Significance:**

Dexamethasone decreased CSF MMP-9 concentrations early in treatment and this may represent one mechanism by which corticosteroids improve outcome in TBM. The strong correlation between CSF MMP-9 and neutrophil count suggests that polymorphonuclear leukocytes may play a central role in the early pathogenesis of TBM.

## Introduction

Tuberculous meningitis (TBM) is the most feared presentation of extra-pulmonary tuberculosis (TB) because about a third of all patients die from disease. We and others have previously shown that adjunctive dexamethasone, administered with anti-tuberculosis drugs, improved the outcome of adults with TBM [1], but the mechanism underlying this effect is not understood. Dexamethasone did not have any significant effect on cerebrospinal (CSF) white cell infiltration or cytokine expression in 93 patients recruited to the clinical trial [2]. A subset of patients had serial brain magnetic resonance imaging, which suggested dexamethasone might decrease the incidence of hydrocephalus and infarction [3]. In children steroids have been shown to reduce CSF protein and cause a more rapid normalization in CSF glucose over placebo [4]. However, no study has demonstrated the mechanism by which dexamethasone reduced case-fatality from TBM.

We hypothesized that dexamethasone improved outcome from TBM by altering the intra-cerebral expression of MMP and the tissue inhibitors of MMPs (TIMPs). MMPs are important mediators of extracellular matrix degradation and are implicated not only in inflammatory central nervous system (CNS) diseases such as multiple sclerosis, HIV dementia and Alzheimer's disease but also in TB [5]–[7]. The blood brain barrier (BBB) is rich in type IV collagen, a substrate of MMP-9 (gelatinase B), and its breakdown is a key initial step in the pathophysiology of CNS leukocyte influx [8], [9]. We and others have shown that CSF concentrations of MMP-9 are elevated in all forms of meningitis and CSF MMP-9 concentrations (corrected for CSF white cell count) were significantly associated with fatal TBM and the extent of cerebral tissue damage [10], [11]. We found that IFN-γ synergistically increases MMP-9 secretion from astrocytes, the most abundant CNS cell and a key component of the BBB [12]. Mouse models of pyogenic bacterial meningitis demonstrate MMP-9∶TIMP-1 ratios are important predictors of tissue destruction, although MMP-9 may also have a significant role in host defense [13], [14]. Our cellular studies on TB-infected macrophages implicate MMP-1 (collagenase-1), -3 (stromelysin-1), -7 (matrilysin) and -10 (stromelysin-2) as critical in the host response to TB [15].

In this study, we measured serial CSF concentrations of a number of MMPs/TIMPs identified as key in TB in a sub-set of adults with TBM recruited to a randomized, placebo-controlled trial of adjunctive dexamethasone [1]. Our aim was to investigate the relationship between dexamethasone treatment, CSF MMP/TIMP expression, and clinical outcome.

## Results

### Comparison of baseline variables

We have compared the baseline clinical features of those included in the MMP study (n = 37) with the rest of the proven HIV uninfected patients recruited to the controlled trial (n = 400) (table 1). Comparison of the patients who received placebo or dexamethasone in the MMP study revealed only CSF opening pressure was significantly different between the groups. The patients were well-balanced with regard to the most important prognostic variables (MRC grade and coma score). Comparison of the patients in the MMP study with the rest of the HIV uninfected patients recruited to the trial revealed some important similarities and differences. Clinical assessments of disease severity (by MRC grade and Glasgow coma score) were not significantly different between the two study groups. However, the patients not included in the MMP study were significantly older and lighter, had lower numbers of white cells in the CSF, and had worse outcomes (table 1).

**Table 1:** Comparison of the baseline clinical features from patients in the study of MMPs with all other HIV uninfected patients recruited to the controlled trial of dexamethasone.

	MMP study sub-group	MMP study sub-group	All other HIV uninfected patients in dexamethasone trial	P-value b
Variable a	Placebo	Dexamethasone		
**Number (n)**	19	18	400	
**Age (yrs)**	31 (23.0–44.0)	25 (19.3–40.5)	40 (29–58)	0.001
**Gender (M∶F) n**	7∶12	8∶10	13∶10	0.098
**TBM grade n (%)**				
**I**	2 (10.5)	6 (33.3)	142 (35.5)	
**II**	11 (57.9)	7 (38.9)	180 (45.0)	0.224
**III**	6 (31.6)	5 (27.8)	78 (19.5)	
**Weight mean (sd–kg)**	49.3 (8.5)	48.4 (6.5)	45.0 (40.0–50.0)	0.004
**Temperature ^o^C**	38.7 (38.0–39.3)	39.0 (38.0–39.5)	38.0 (37.5–39.0)	0.001
**GCS at presentation**	13 (10–14)	13.5 (10–15)	14 (12–15)	0.210
**Duration of symptoms (days)**	17 (12–21)	17 (12–26.25)	15 (10–30)	0.617
**Cranial Nerve Palsy n (%)**	8 (42.1)	6 (33.3)	115 (28.8)	0.353
**Hemiparesis n (%)**	2 (10.5)	3 (16.7)	59 (14.8)	0.552
**Paraparesis n (%)**	0 (0)	1 (5.6)	33 (8.3)	0.241
**Opening pressure (cm)** c	18 (16–32)	33 (19–39)	22 (13–39)	0.478
**Total WCC (cells/µl)**	470 (130–625)	250 (147–613)	104 (27–251)	<0.001
**Neutrophils (%)**	30 (18–43)	40 (24.5–67.5)	5 (0–20.0)	<0.001
**Lymphocytes (%)**	70 (57–82)	60 (42–75.5)	95 (80.0–100)	<0.001
**Protein (g/L)**	1.6 (1.0–3.0)	2.0 (1.5–3.0)	1.4 (0.8–2.2)	0.020
**Lactate (mmol/L)**	6.2 (4.6–8.1)	6.6 (5.2–8.9)	5.1 (3.6–6.9)	0.024
**Glucose ratio–CSF/blood**	0.17 (0.09–0.30)	0.25 (0.14–0.34)	0.29 (0.19–0.39)	0.004
**Albumin ratio–CSF/blood (%)**	1.12 (0.58–2.84)	2.03 (0.65–2.73)	NA d	NA d
**Death n (%)**	3 (15.8)	2 (11.1)	120 (30.0)	0.016
**Combined poor outcome n (%)**	4 (21.1)	4 (22.2)	171 (42.8)	0.006

a Values are median (IQR) or number (percentage) unless stated otherwise.

b Comparison between the variables of patients in the MMP study with all other patients recruited to the controlled trial (continuous variables compared by the Mann-Whitney U test; categorical variables by the Chi square test).

c Comparison of baseline variables between patients given placebo or dexamethasone in the MMP study showed only CSF opening pressure was significantly different (P<0.05) between the groups.

d Data not available in this group.

185 CSF specimens were taken from the 37 patients included in the MMP study. 141 (76%) specimens were available for analysis of longitudinal changes in MMP/TIMP concentrations: 66/94 (70%) were from placebo and 75/91 (82%) from dexamethasone group patients (a mean of 3.8 (+/−1.2) samples per patient).

### Baseline CSF MMP and TIMP concentrations

Baseline pre-treatment concentrations of MMPs and TIMPs are summarized in table 2 and are similar between the two groups with the exception of TIMP-2, which was significantly higher in the patients give placebo. MMP-2, -3 and -9 concentrations were detectable in all 30 pre-treatment CSF samples analyzed and MMP-8 concentrations were detectable in 29 (97%). CSF concentrations of MMP-1 and -7 were only detectable in 9 (30%) patients and MMP-10 in 10 (33%) patients. All 30 patients had detectable TIMP-1 and -2 and 29 (97%) patients had detectable CSF TIMP-4.

**Table 2:** Baseline CSF MMP/TIMP concentrations.

Variable*	Placebo	Dexamethasone
**Number (n)**	15	15
**MMP-1 (ng/ml)**	0.13 (0.13–0.49)	0.13 (0.13–0.24)
**MMP-2 (ng/ml)**	47.0 (37.5–53.2)	42.2 (33.3–48.7)
**MMP-3 (ng/ml)**	1.22 (0.80–1.46)	0.97 (0.27–1.66)
**MMP-7 (ng/ml)**	0.078 (0.078–0.079)	0.078 (0.075–0.12)
**MMP-8 (ng/ml)**	18.9 (10.9–28.4)	12.6 (7.9–28.6)
**MMP-9 (ng/ml)**	108.3 (37.8–226.5)	110.8 (93.4–269.2)
**MMP-10 (ng/ml)**	0.04 (0.04–0.11)	0.04 (0.04–0.11)
**TIMP-1 (ng/ml)**	248.6 (116.2–421.1)	180.1 (83.9–241.0)
**TIMP-2 (ng/ml)** #	31.6 (28.2–38.6)	23.0 (20.9–29.6)
**TIMP-4 (ng/ml)**	0.43 (0.32–0.47)	0.49 (0.32–0.87)

* Values are median (IQR) unless stated otherwise.

# p = 0.01 for group comparison.

### Dexamethasone specifically reduces CSF MMP-9 concentration at day 5 of TBM treatment

**Figure 1:**
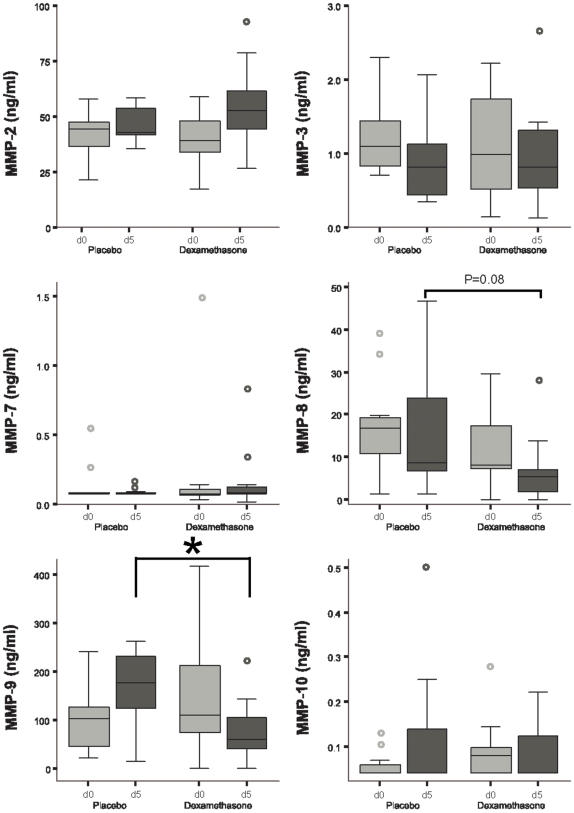
Dexamethasone reduces CSF MMP-9 concentrations in early follow up samples. Paired CSF samples from patients with tuberculous meningitis were taken pre-treatment and at early follow up, a median 5 days (range 3–8) into anti-tuberculosis therapy, and analyzed for MMP-1, -2, -3, -7, -8, -9 & -10 concentration by ELISA (MMP-6, -7 & -8 do not exist). Patients who received placebo (light grey bars, n = 11) had significantly higher MMP-9 concentrations in their early follow-up CSF samples than those who received dexamethasone (dark grey bars, n = 11). *, p = 0.01. The black line represents the median value and the box the interquartile range. Whiskers represent 1.5 times the interquartile range away from the box.

**Figure 2:**
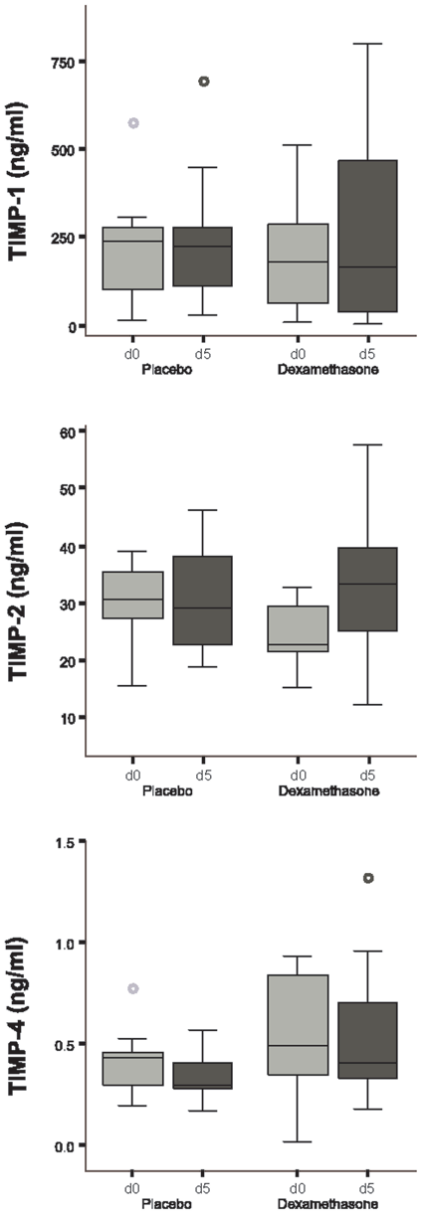
CSF TIMP concentrations in early follow up samples are not affected by dexamethasone. Paired CSF samples from patients with tuberculous meningitis were taken pre-treatment and at early follow up, a median 5 days (range 3–8) into anti-tuberculosis therapy, and analyzed for TIMP-1, -2, & -4 concentrations by ELISA. The black line represents the median value and the box the interquartile range. Whiskers represent 1.5 times the interquartile range away from the box.

22 patients had both a pre-treatment and at least one paired ‘early’ follow up sample available for analysis, taken a median 5 days (range 3–8) into anti-tuberculosis treatment, equally spilt between placebo and dexamethasone groups. Only CSF MMP-9 concentrations were significantly lower in the early follow-up samples of patients given dexamethasone compared to placebo (p = 0.01, figure 1), confirmed by repeated measures ANOVA with CSF white cell count as a covariate (p = 0.002). 3/11 (27%) patients in the placebo group had a fall in their CSF MMP-9 concentration compared with 9/11 (82%) of the dexamethasone group between pre-treatment and the early follow-up sample (p = 0.03). However, we could not find a significant relationship between those patients whose CSF MMP-9 concentration fell and outcome; 1/12 (8%) patients with a fall in CSF MMP-9 died and 1/10 (10%) died in the non-faller group, the data were similar for death or disability (data not shown). There was a similar but less marked effect of dexamethasone on MMP-8 concentrations. A trend for lower CSF MMP-8 concentrations in the dexamethasone arm (p = 0.08) was not significant in the repeated measures ANOVA. TIMPs are the natural inhibitors of MMPs but we were unable to find any effect of dexamethasone on changes in CSF TIMP concentration in the same early follow-up (day 5) samples (figure 2).

MMP-9 concentrations at later time points were not statistically different between the two groups. At day 30 CSF MMP-9 concentration was 252.6 ng/ml (IQR 154.6–298.6) in the placebo group and 249.9 ng/ml (IQR 67.5–406.6) in the dexamethasone group. At day 60 CSF MMP-9 concentration was 281.6 ng/ml (IQR 94.1–345.0) in the placebo group and 181.9 ng/ml (IQR 101.8–300.7) in the dexamethasone group. Day 270 concentrations for all CSF MMP/TIMP and their statistical change from baseline are summarized in table 3. Of note MMP-3, -8, -9 and TIMP-1 fell and TIMP-4 rose significantly over the course of 9 months anti-tuberculosis treatment. No other significant relationships were found at any time points for any other MMPs and treatment group. MMP/TIMP concentrations at all time points for both groups have been summarized in supplementary table S1.

**Table 3:** Day 270 CSF MMP/TIMP concentrations (n = 19).

Variable	CSF Concentration [median (IQR)]*	P value#
**MMP-1 (ng/ml)**	0.13 (0.13–0.13)	0.34
**MMP-2 (ng/ml)**	36.7 (33.0–50.7)	0.26
**MMP-3 (ng/ml)**	0.26 (0.12–0.45)	<0.001
**MMP-7 (ng/ml)**	0.08 (0.07–0.11)	0.63
**MMP-8 (ng/ml)**	0.03 (0.03–0.03)	<0.001
**MMP-9 (ng/ml)**	4.7 (0.9–12.2)	<0.001
**MMP-10 (ng/ml)**	0.04 (0.04–0.05)	0.20
**TIMP-1 (ng/ml)**	23.9 (15.2–36.0)	<0.001
**TIMP-2 (ng/ml)**	26.4 (23.1–37.4)	0.82
**TIMP-4 (ng/ml)**	2.1 (1.4–3.1)	<0.001

* Data are presented unstratified for treatment group.

# Comparison to baseline value in table 2.

### The relationship between CSF WCC and MMP-9 concentrations

**Figure 3:**
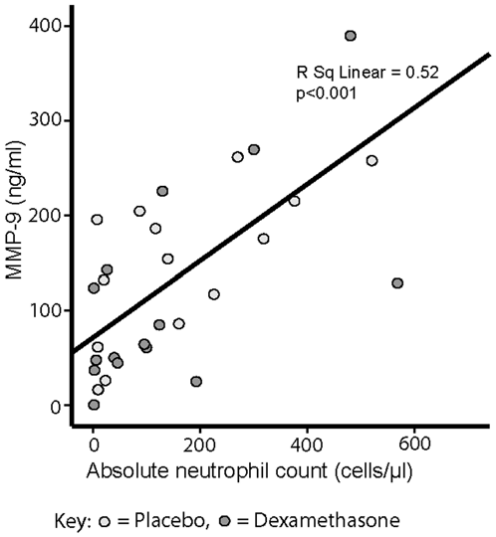
CSF MMP-9 concentration and CSF neutrophil count are significantly correlated in early follow up samples. Early follow-up CSF samples, taken a median of 5 days (range 3–8, n = 29) into anti-tuberculosis treatment, from patients with tuberculous meningitis were analyzed for correlation between absolute neutrophil count and MMP-9 concentration (measured by ELISA). The R^2^ value for the correlation was 0.52 (p<0.001). Patients who received placebo are represented by light grey circles and dexamethasone by dark grey circles.

Neutrophils play a key but possibly under-recognized role in host defense to mycobacteria and release preformed MMP-9 from granzymes to cross the BBB [16], [17]. CSF MMP-9 concentration and neutrophil count were strongly correlated on analysis of all 141 samples (R^2^ = 0.19, p<0.001). There was a significant correlation between MMP-9 concentration and CSF WCC (R^2^ = 0.31, P = 0.004) in all 29 available early follow-up (day 5) samples and a stronger correlation with CSF neutrophil count (Figure 3, R^2^ = 0.52, p<0.001) which was dexamethasone independent. CSF neutrophil count was not independently related to outcome.

### The relationship between MMP-9 and other CSF inflammatory parameters

BBB disruption is potentially related to MMP activity [18]. There were significant correlations between CSF total protein (the only CSF analyte to fall significantly due to dexamethasone [2]) and MMP-9 (R^2^ = 0.18, p<0.001) concentrations but we could find no relationship between the albumin index (a marker of BBB permeability) and CSF MMP/TIMP concentration. At the day 5 time point MMP-8 and -9 were closely correlated (R^2^ = 0.67, p<0.001, n = 29) but there was only a weak relationship between MMP-9 and its specific inhibitor TIMP-1 (R^2^ = 0.13, p<0.05, n = 29). There were no other relationships between MMP-9 and any of the other MMP/TIMP concentrations at this time point.

We investigated the relationship between CSF concentrations of TNF-α, IFN-γ, IL-6, IL-8, IL-10 (data not presented) and MMP-9 in all 141 specimens and found only IL-10 and MMP-9 to be correlated (R^2^ = 0.22, p<0.001). Concentrations of IL-1β and IL-12p70 levels were below the level of detection in all but 6 and 8 specimens respectively so were not analyzed. Specific examination of pre-treatment and early follow-up samples did not reveal any other significant relationships.

### Analysis of all baseline clinical and laboratory variables and relationship to baseline clinical severity and treatment outcome measurements

We investigated the relationship between increasing severity of coma (i.e. GCS of <15 or not on admission to hospital) and clinical and laboratory variables including CSF MMP/TIMP concentrations by logistic regression. In univariate analysis only gender and CSF chloride concentration went forward to entry into the final model, but neither remained significant.

We then performed univariate analysis to look for relationships between combined poor outcome (death and disability) and clinical and laboratory findings. Gender, baseline GCS, cranial nerve palsy and hemiparesis on presentation went forward into multivariate analysis where only hemiparesis remained significant in the final model (OR = 0.05, p = 0.04 [95% CI 0.003, 0.92]).

We found no other significant relationships between pre-treatment MMP/TIMP concentrations (n = 30) and mortality, cranial nerve palsy, hemiparesis and paraparesis on admission by multivariate analysis, although there was a significant relationship between lower pre-treatment MMP-2 concentrations in CSF and hemiparesis (OR = 0, p = 0.02 [95% CI 0, 0.24]) on univariate analysis. There was no relationship between duration of symptoms and MMP/TIMP concentrations.

## Discussion

In this study we have investigated the influence of dexamethasone on the CSF secretion of MMPs/TIMPs in (n = 37) adults with TBM. Despite the small number of patients we found dexamethasone significantly decreased CSF MMP-9 concentrations early in the course of TBM treatment and thus may represent one mechanism by which corticosteroids improve outcome. As dexamethasone did not influence the CSF concentrations of other MMPs this suggests a specific mode of action. Our previous investigations failed to demonstrate a significant effect of dexamethasone on serial CSF cytokines or chemokines, although CSF protein fell faster in the dexamethasone treated group and there was a trend toward lower IFN-γ concentrations [2]. These data are consistent with our *in vitro* data that indicate IFN-γ influences intra-cerebral MMP-9 secretion[12].

MMP-9 is quantitatively the most significant MMP released by macrophages and concentrations are increased in lungs and pleural fluid of TB patients [19], [20]. Serum MMP-9 concentrations may be raised and associated with pulmonary disease severity [21]. *In vitro*, MMP-9 is secreted from monocyte cell lines in response to stimulation by a number of TB components such as lipoarabinomannan (LAM), a process dependent upon the transcription factor AP-1 [19], [22]. Previous studies suggest MMP-9 may be an important molecule in TBM pathogenesis. A study of adults with TBM, viral, and bacterial meningitis found increased CSF MMP-9 concentrations were associated with more severe disease and death in all cases [11]. A study of TBM alone has replicated this finding [10]. Other cellular work demonstrates that *M.tb*-infected monocytes drive a network involving IFN-γ, TNF-α, IL-1β and NFκB resulting in increased astrocyte MMP-9 secretion without altered TIMP-1/2 secretion indicating a role for glial cells in the development of the matrix degrading phenotype present in CNS TB [12], [23]. However, murine studies suggest MMP-9 is also important in host defense to *M. tuberculosis*. Macrophages from mice resistant to *M. tuberculosis* produce more MMP-9 mRNA in response to intracellular infection than susceptible mice [24]. Non-specific MMP inhibition blocked early dissemination of *M. tuberculosis* and MMP-9-knockout mice had reduced pulmonary recruitment of macrophages and poorer granuloma formation [25]. Therefore *in vivo* a balance may exist between the MMP-9 necessary for host-defense and excess causing the unwanted effects opposed by dexamethasone.

Our data suggest that the majority of early CSF MMP-9 is released from neutrophils as there was a strong correlation between both baseline values. Other studies have reported strong correlations between MMP-9 and neutrophils in viral meningitis and with total white cells in TBM [26], [27]. Neutrophils release large amounts of preformed MMP-9 in response to many inflammatory stimuli, which may explain the lack of correlation observed between CSF MMP-9 and the pro-inflammatory cytokines measured [28]. Additionally there was a trend in our data to suggest that MMP-8, a collagenase principally derived from neutrophils and important in bacterial meningitis, might also be decreased by dexamethasone [16], [29]. The role of the neutrophil in the pathogenesis of TB is not well understood. Peripheral neutrophil counts have been inversely related to risk of TB in contacts of infectious cases and neutrophil depletion *ex vivo* impaired the ability of whole blood to restrict mycobacterial growth via depletion of secreted anti-microbial peptides [17]. A report that neutrophil derived anti-microbial peptides are released in response to *M. tuberculosis* infection but not expressed in the tuberculous granuloma is consistent with the proposed early role for neutrophils in TB [22]. In addition, adults with TBM and a high proportion of neutrophils in the CSF have an increased chance of survival [30].

Given the strong correlation between CSF neutrophils and MMP-9 concentrations we investigated the influence of dexamethasone on CSF neutrophil numbers, but did not observe any differences between the treatment groups at any time point. However, dexamethasone may affect function rather than absolute numbers. Corticosteroids can rapidly prevent neutrophil degranulation independently of effects on protein synthesis [31]. The restricted release of neutrophil-derived proteins in dexamethasone treated patients may have contributed to the significant reduction in CSF protein observed in the dexamethasone group in the published study involving a larger number of patients from the parent trial [2].

The study has a number of limitations. First, we only studied a small sub-group of patients recruited to the controlled trial. In addition we only studied time points at 0 and 5 days that lead to our conclusions, further work must delineate more clearly when dexamethasone is having this early effect. We could only study patients from one centre, with bacteriologically confirmed disease, in whom multiple serial CSF specimens were available. Therefore because of its small size the study lacked power to detect small effects of dexamethasone and to link effects with outcome. In addition, the patients selected may not be representative of all the patients in the trial. However, the baseline clinical variables most strongly associated with outcome – disease severity grade and coma score - were well matched, both within the patients included in the MMP study and when these patients were compared with the rest of the trial patients. The worse outcomes in patients not included in the MMP study may be partly explained by their older age and lower numbers of white cells in the CSF, both independent risk factors for death from TBM [1]. Also, we used ELISAs that detected both active and pro-MMP-9 and thus could not differentiate between biologically active and inactive or degraded MMP-9 [32]. Differences in the activity of MMP-9 (assessed by zymography) have been reported in experimental pyogenic meningitis where treatment with antibiotics alone increased CSF MMP-9 activity, but dexamethasone co-administration suppressed this effect [33].

In summary, this investigation has shown that dexamethasone may influence outcome from TBM by reducing MMP-9 secretion. However, future research needs to delineate the roles of neutrophils and their MMPs, specifically MMP-8 and -9, in the early stages of TBM treatment more clearly. Targeting neutrophil-derived, MMP-driven, inflammatory responses early in TBM may be a potential therapeutic strategy.

## Methods

### Study participants and setting

We studied a subset of patients from a previously reported randomized, double-blind, placebo-controlled trial of adjunctive dexamethasone for the treatment of TBM in 545 Vietnamese adults [1]. To be eligible for selection for the current study patients had to have been recruited at the Hospital for Tropical Diseases (HTD), Ho Chi Minh City, have a microbiologically confirmed diagnosis of TBM (*Mycobacterium tuberculosis* cultured from the CSF), negative HIV-1 & 2 antibodies, and at least two serial CSF specimens taken within the first 60 days of treatment (when dexamethasone was stopped) available for analysis, unless they had died within this period.

Disease severity at admission was defined by the Glasgow coma scale (GCS). Assessment of GCS was performed by summation of clinical assessment of eye opening (awarded 1–4 points), best verbal response (1–5 points) and best motor function (1–6 points) where lower values indicate more severe neurological dysfunction [34]. Disease severity was also assessed using the United Kingdom Medical Research Council grade (UK MRC) for TBM [34]: patients in grade I had a GCS of 15 (fully conscious) and no focal neurological signs; patients in grade II had a GCS of between 11 and 14 and/or focal neurological signs; and patients in grade III had severe coma with a GCS of less than 11.

The ethical and scientific committees of Imperial College, Oxford University and the Hospital for Tropical Diseases approved the study and written, informed consent to participate in the study was obtained from the patients or their relatives. All clinical investigation was conducted according to the principles expressed in the Declaration of Helsinki.

### Treatment and specimen collection

Adults were allocated randomly to start dexamethasone sodium phosphate or placebo as soon as possible after the start of four drug anti-tuberculosis therapy as previously described [1]. Briefly, adults with grade II or III disease received intra-venous study drug for 4 weeks (week 1 0.4 mg/kg/day); week 2 0.3 mg/kg/day, week 3 0.2 mg/kg/day, week 4 0.1 mg/kg/day), then 4 weeks of tablets starting at 4 mg total dose per day, reducing by 1 mg per week until zero. Those with grade I disease received 2 weeks of intra-venous study drug (week 1 0.3 mg/kg/day, week 2 0.2 mg/kg/day) then 4 weeks of tablets (week 3 0.1 mg/kg/day; then 3 mg total per day reducing by 1 mg per week until zero). Serial CSF samples (frozen at −70°C) and routine clinical investigations were collected as part of normal clinical care pre-treatment and on study days 3, 7, 30, 60 and 270.

### Assessment of the clinical and CSF inflammatory response to treatment

The primary outcome of the trial was death or severe disability 9 months after randomization and was assessed blind to the treatment allocation [1]. Likewise, treatment allocation was unknown to those assessing conventional and experimental CSF inflammatory responses including MMPs/TIMPs. CSF white cell count, protein, glucose and lactate concentrations were determined by standard methods. Blood brain barrier integrity was assessed by measurement of paired CSF and plasma albumin concentrations by standard methods. The albumin index was calculated using the formula [albumin*_csf_*]/[albumin*_plasma_*] [30]. The concentrations of CSF cytokines (IFN-γ, TNF-α, and the interleukins -1β, -6, -8, -10, and 12p70) were determined by cytometric bead array assay (BD Biosciences) as described [2].

### CSF MMP and TIMP concentration measurement

MMP-1, -2, -3, -7, -8, -9 and -10 and TIMP-1, -2 and -4 were measured by ELISA (R&D, Abingdon, UK) according to the manufacturer's instructions. The lower level of sensitivity for the ELISAs were 156 pg/ml for MMP-1 and -7, 78 pg/ml for MMP-2 and -10, and 31 pg/ml for MMP-3, -8, -9, TIMP-1, -2 and -4. Samples with high MMP/TIMP concentrations were diluted as appropriate. Values below the level of detection were assigned an arbitrary value halfway between 0 and the lower level of sensitivity.

### Statistical analysis

The analysis followed a pre-defined plan, unless otherwise stated as exploratory analysis. Continuous variables were compared by Student's t-test if normally distributed and Mann-Whitney U test if non-normally distributed. Where more than two groups were compared the Kruskal-Wallis test was used for non-parametric data. Categorical data were compared using Fisher's exact test or the Χ^2^ test. All p-values were two sided and a value of <0.05 was taken as significant. The changes in MMP/TIMP concentration between pre-treatment and follow-up samples were calculated using repeated measures ANOVA with dexamethasone as a covariate. Baseline MMPs and TIMPs were analyzed by univariate analysis to examine relationships between presenting clinical features and the specific outcomes of death and combined poor outcome (defined in the original trial as death or severe disability at 9 month follow-up). Clinical and laboratory features with P<0.15 were then entered into a multivariate model using forward and backward likelihood ratio logistic regression. All analyses were performed using SPSS version 15.0 (SPSS Corp, Chicago, IL, US).

## Supporting Information

Table S1 Summary of CSF MMP/TIMP concentrations at all time points. # Data for all patients presented, paired analysis presented in text.
(0.05 MB DOC)
