# Supplementary material for: Dexamethasone, Cerebrospinal Fluid Matrix Metalloproteinase Concentrations and Clinical Outcomes in Tuberculous Meningitis
Source: PLoS One. 2009 Sep 30;4(9):e7277. doi: 10.1371/journal.pone.0007277 (PMC2748711; doi:10.1371/journal.pone.0007277)
Supplement: Table S1 — Summary of CSF MMP/TIMP concentrations at all time points. # Data for all patients presented, paired analysis presented in text. (0.05 MB DOC) [file pone.0007277.s001.doc]

| **Analyte** | **Day 0** | **Day 0** | **Day 5^#^** | **Day 5^#^** | **Day 30** | **Day 30** | **Day 60** | **Day 60** | **Day 270** | **Day 270** |
| --- | --- | --- | --- | --- | --- | --- | --- | --- | --- | --- |
|  | **Placebo** | **Dex** | **Placebo** | **Dex** | **Placebo** | **Dex** | **Placebo** | **Dex** | **Placebo** | **Dex** |
| **MMP-1** | 0.13  (0.13-0.49) | 0.13  (0.13-0.24) | 0.13  (0.13-0.13) | 0.13  (0.13-0.13) | 0.13  (0.13-0.14) | 0.13  (0.13-0.13) | 0.13  (0.13-0.13) | 0.13  (0.13-0.13) | 0.13  (0.13-0.17) | 0.13  (0.13-0.13) |
| **MMP-2** | 47.0  (37.5-53.2) | 42.2  (33.3-48.7) | 46.7  (41.2-58.4) | 50.0  (43.3-63.1) | 88.1  (64.5-101.5) | 72.9  (61.6-89.8) | 60.3  (56.3-98.2) | 76.6  (58.3-98.3) | 39.7  (33.8-62.8) | 33.1  (27.7-44.5) |
| **MMP-3** | 1.22  (0.80-1.46) | 0.97  (0.27-1.66) | 0.95  (0.42-1.13) | 0.94  (0.50-1.36) | 0.60  (0.39-0.89) | 0.62  (0.25-0.99) | 0.25  (0.20-0.48) | 0.36  (0.19-0.67) | 0.26  (0.20-0.54) | 0.25  (0.08-0.59) |
| **MMP-7** | 0.078  (0.078-0.079) | 0.078  (0.075-0.12) | 0.078  (0.078-0.078) | 0.078 (0.078-0.13) | 0.1  (0.078-0.23) | 0.078  (0.078-0.082) | 0.078  (0.078-0.25) | 0.078  (0.078-0.12) | 0.078  (0.078-0.14) | 0.078  (0.078-0.14) |
| **MMP-8** | 18.9  (10.9-28.4) | 12.6  (7.9-28.6) | 17.2  (6.1-27.4) | 5.5  (1.67-26.3) | 6.9  (0.65-15.4) | 4.1  (0.05-23.0) | 2.3  (0.03-9.3) | 0.9  (0.03-3.0) | 0.03  (0.03-0.03) | 0.03  (0.03-0.03) |
| **MMP-9** | 108.3  (37.8-226.5) | 110.8  (93.4-269.2) | 180.6  (93.6-247.0) | 74.4  (45.2-204.8) | 252.6  (154.6-298.6) | 249.9  (67.5-406.6) | 281.6  (94.1-345.0) | 181.9  (101.8-300.7) | 11.3  (0.9-25.1) | 3.8  (0.5-7.9) |
| **MMP-10** | 0.04  (0.04-0.11) | 0.04  (0.04-0.11) | 0.05  (0.04-0.15) | 0.04  (0.04-0.15) | 0.09  (0.04-0.20) | 0.04  (0.04-0.13) | 0.04  (0.04-0.07) | 0.04  (0.04-0.04) | 0.04  (0.04-0.04) | 0.04  (0.04-0.12) |
| **TIMP-1** | 248.6  (116.2-421.1) | 180.1  (83.9-241.0) | 236.2  (115.3-415.9) | 195.9  (52.9-524.7) | 69.9  (39.6-225.7) | 124.1  (67.6-183.1) | 75.2  (26.9-172.3) | 97.8  (28.4-272.6) | 22.7  (15.0-35.1) | 34.3  (18.9-48.5) |
| **TIMP-2** | 31.6  (28.2-38.6) | 23.0  (20.9-29.6) | 37.0  (23.5-42.6) | 34.5  (26.7-44.9) | 37.3  (31.8-66.3) | 32.6  (25.1-39.5) | 41.2  (31.6-46.7) | 36.6  (31.8-48.0) | 33.6  (23.4-50.4) | 26.1  (19.6-30.8) |
| **TIMP-4** | 0.43  (0.32-0.47) | 0.49  (0.32-0.87) | 0.35  (0.28-0.72) | 0.38  (0.29-0.72) | 0.36  (0.29-0.48) | 0.53  (0.36-0.88) | 0.82  (0.48-0.97) | 0.88  (0.53-1.21) | 2.04  (1.22-3.07) | 2.31  (1.52-3.16) |
| **N=** | 15 | 15 | 16 | 16 | 12 | 13 | 11 | 11 | 10 | 9 |

Table S1: Summary of CSF MMP/TIMP concentrations at all time points.

# Data for all patients presented, paired analysis presented in text.
